# Changes in the Folate Content and Fatty Acid Profile in Fermented Milk Produced with Different Starter Cultures during Storage

**DOI:** 10.3390/molecules26196063

**Published:** 2021-10-07

**Authors:** Marta Czarnowska-Kujawska, Beata Paszczyk

**Affiliations:** Department of Commodity and Food Analysis, The Faculty of Food Sciences, University of Warmia and Mazury in Olsztyn, 10-726 Olsztyn, Poland; paszczyk@uwm.edu.pl

**Keywords:** dairy products, biofortification, probiotic, LAB, folic acid, fatty acids, CLA, storage time

## Abstract

The application of bacterial cultures in food fermentation is a novel strategy to increase the “natural” levels of bioactive compounds. The unique ability of lactic acid bacteria (LAB) to produce folate, B vitamins, and conjugated linolenic acid *cis*9*trans*11 C18:2 (CLA) during cold storage up to 21 days was studied. Although some species of LAB can produce folates and other important nutrients, little is known about the production ability of yogurt starter cultures. Pasteurized milk samples were inoculated with four different combinations of commercially available yogurt vaccines, including starter cultures of *Bifidobacterium bifidum*. Both the type of vaccine and the time of storage at 8 °C had a significant effect on the folate and CLA contents in the tested fermented milks. The highest folate content (105.4 µg/kg) was found in fresh fermented milk inoculated with *Lactobacillus delbrueckii*, *Streptococcus thermophilus*, and *Bifidobacterium bifidum.* Only the mix of *Lactobacillus delbrueckii* subsp. *bulgaricus*, *Streptococcus thermophilus*, and *Bifidobacterium bifidum* showed potential (59% increase) to synthesize folate during seven days of storage. A significant increase in the content of CLA, when compared to fresh fermented milk, was observed during cold storage for up to 21 days in products enriched with *Bifidobacterium bifidum*.

## 1. Introduction

Improving the nutritional value of food is one of the major challenges for the food industry of the twenty-first century. Consumers, apart from new tastes and flavors, are increasingly interested in the origin and bioactive properties of foods that can affect their health and wellbeing [1,2]. Folates are reduced folic acid derivatives (so-called polyglutamates) which naturally occur in food, both of plant and animal origin. The name folic acid refers only to the synthetic form of the vitamin, which is commonly used for food fortification and supplementation [1,3]. Folates cannot be synthesized by humans and must be obtained exogenously. The richest sources are animal liver, yeast, wheat germ, and green leafy vegetables, such as spinach, broccoli and asparagus, and pulses [4]. Folates belong to the water-soluble B vitamins group and are essential components of the human diet for the synthesis reaction of nucleotides and vitamins. They are the source of methyl groups in the process of homocysteine remethylation to methionine [1,5]. Despite such an important role, acquired folate deficiency is common and affects billions of people worldwide, both in developing and developed countries and in different age groups. This micronutrient deficiency is associated with poor diet, malabsorption, alcohol consumption, obesity, and kidney failure [1,4]. An increased risk of folate deficiency especially concerns the elderly (because of lower food intake), children (who are likely to consume a limited variety of food), and pregnant women (because of the critical role of folates in fetus neural tube development). Low folate intake increases the risk of birth defects (spina bifida, anencephaly, encephalocele) [4]. Prolonged insufficient folate consumption is also linked to macrocytic anemia, cardiovascular diseases, neurodegenerative diseases (Alzheimer’s, Parkinson’s), certain forms of cancers (colorectal, breast, cervical, lung, pancreatic cancer), and even an increased risk of depressive mental disorders [6,7].

In the face of insufficient folate consumption, many developed countries, including the U.S. and Canada, have introduced mandatory folic acid fortification of wheat and/or maize flour at the industrial level to increase the folate intake of the whole population [8,9]. For pregnant women, daily folic acid supplementation is recommended to reduce the risk of low birth weight and congenital malformations, including neural tube defects [10]. However, food fortification programs are not always effective. It is estimated currently that no more than 25% of folic-acid-preventable neural tube defects are actually prevented [9]. Moreover, higher levels of synthetic folic acid in the diet can cause adverse effects, such as masking a vitamin B12 deficiency, which may result in the progression of neuropathy to an irreversible point [11]. Moreover, high physiological folate concentrations and folate overload, as a result of excessive supplementation with folic acid, may increase the risk of impaired brain development in embryogenesis and even possess a growth advantage for pre-cancerous altered cells [4,5]. Therefore, due to the potential risk of using synthetic folic acid, but also limited availability of folate-rich foods (depending on the season and geographic and economic factors), food fortification by natural folate is a necessary alternative [9].

One novel strategy to increase natural folate levels is the application of bacterial cultures in food fermentation. Fermented milk, especially yogurt, is the appropriate choice and ideal matrix for bio-fortification for several reasons. Firstly, it contains folate-binding protein, which improves folates stability and bioavailability. Secondly, it is consumed in many countries worldwide and is increasingly popular among consumers, who consider it healthy, with many therapeutic and desirable effects. Finally, fermented dairy products can significantly contribute to the daily recommended levels by in situ fortifications through fermentation with the aid of folate-producing microbes [9,12,13].

Lactic acid bacteria (LAB) are microorganisms involved in the traditional fermentation process. Some of them are considered probiotic species, which are beneficial to their hosts in many ways and are also known for folate production in high amounts, which may improve the nutritional composition of fermented products [5,14]. The ability of *Streptococcus thermophilus* and *Lactococcus lactis* to synthesize folate has been reported, and increased folate production by *Lactobacillus plantarum* has also been demonstrated. Unfortunately, in the milk fermentation process, the majority of the bacteria are folate utilizers and decrease folate amounts. Therefore, only the proper selection of suitable starter culture or a consortium of folate producers would allow increasing the dietary folate content [12,14,15,16,17]. For this reason, further research and development of folate-producing bacteria for food applications should be encouraged.

Besides the ability to produce folates, probiotic bacteria also show the ability to synthesize conjugated linolenic acid *cis*9*trans*11 C18:2 (CLA). This acid is the main representative of the conjugated dienoic milk fat group and constitutes from 75% to over 90% of the sum of these isomers in the fat of milk and dairy products [18,19,20]. As reported by various authors [21,22,23,24,25], CLA displays a number of health-positive properties, e.g., anticarcinogenic, anti-atherosclerotic, antioxidative, and anti-inflammatory effects. The content of CLA in milk fat may vary widely depending on many factors, such as animal feeding, breed, age, and lactation period [26,27,28,29,30,31,32]. In dairy products, it may also be affected by the production process. According to some studies [33,34,35,36,37,38], technological treatments applied in the industry and additives used may influence CLA content in the fatty acid composition of dairy products. According to literature data [39,40,41,42,43,44], selected strains of bacteria are capable of CLA synthesis during fermentation. As reported by Kim and Liu [40], CLA content in fermented milk is affected by the type of bacterial strain applied, cell count, appropriate concentration of the substrate, and incubation conditions (time and pH).

In the food additives market, special mixtures of starter cultures that provide good conditions for the right milk fermentation are commercially available. These are mixtures of one or more strains with acid-forming, protective, and organoleptic properties. Producers are expanding their offer with probiotic strains with other properties that preserve or add flavor and are also popular among consumers. The objectives of this study were to analyze the folate content in fermented milk samples produced with the use of commercially available yogurt vaccines, including starter cultures of *Bifidobacterium bifidum*, and to assess the stability of the produced folate during refrigerated storage. Moreover, in the tested fermented milk, the influence of the used starter cultures and the time of cold storage on the fatty acid profile (especially on the content of conjugated linoleic acid *cis*9*trans*11 C18:2 (CLA)) were evaluated.

## 2. Results and Discussion

### 2.1. Folates

The folate content in milk after pasteurization, which is a basic material for the production of fermented milk beverages, is not very high when compared to other folate-rich foods. Based on the data obtained using three analytical methods (HPLC, radio protein binding, and microbiological), the folate content determined in cow’s milk ranged from 50 to 100 µg/L [5,45,46]. In the authors’ previous study [47], the folate content in the pasteurized cow’s milk was reported to be 36.9 µg/kg. Raw milk processing, such as pasteurization or UHT, as well as transport conditions and storage time, are known to reduce folate levels. Folates are labile compounds, and increased temperature, exposure to oxidizing agents, UV light and sunlight, unfavorable pH, and interaction with other food ingredients (such as metal cations) enhance folate degradation and/or interconversions [16,48]. Dairy products are often fermented by LAB alone or in combination with other microorganisms before consumption [15]. Several studies have reported an increase in folate content in different foods after fermentation by LAB selected for their biosynthesis capacities [45,46,49,50,51,52,53].

In the current study of fermented milk (FM) samples, two folate forms were identified: 5-methyltetrahydrofolate (5-CH_3_-H_4_folate) and tetrahydrofolate (H_4_folate). H_4_folate, 5-CH_3_-H_4_folate, and the total folate content of fresh and stored tested fermented products are shown in Table 1. The methyl form was dominant, which is in agreement with previous studies [5,16,47,49,54]. The lower content of H_4_folate may result from the fact that this vitamer, among other folate vitamers, is likely to oxidize into other folate forms at low pH [9]. *Lactobacillus delbrueckii* subsp. *bulgaricus* and *Streptococcus thermophilus* only accumulated comparable amounts of H_4_folate and 5-CH_3_-H_4_folate in the FM 1, both in fresh material and material stored up to 21 days. In the fresh fermented milk, the H_4_folate content ranged from 12.2 in FM 2 inoculated with *Lactococcus lactis and Leuconostoc* to 27.5 µg/kg in FM 3 inoculated with the combination of *Lactobacillus delbrueckii*, *Streptococcus thermophilus*, and *Bifidobacterium bifidum*. The highest 5-CH_3_-H_4_folate (84.0 µg/kg) was also found in FM 3, but the lowest (24.4 µg/kg) was in FM 1 inoculated with *Lactobacillus delbrueckii* and *Streptococcus thermophilus*.

Similarly, the lowest total folate content (45.3 µg/kg) was observed in FM 1, while the highest (105.4 µg/kg) was in FM 3. With the exception of product 1, in the analyzed samples, content levels were significantly higher than reported previously for pasteurized milk [47]. An increase of 1.6–2.8 fold was observed in fresh fermented milks 2, 3, and 4.

In the current experiment, during refrigerated storage for at least seven days, only the combination of *Lactobacillus delbrueckii* subsp. *bulgaricus*, *Streptococcus thermophilus*, and *Bifidobacterium bifidum* in FM 4 showed the potential to produce one folate vitamer (5-CH_3_-H_4_folate). The increase in the methyl form content was 59%, which resulted in a total folate content of 79.2 µg/kg on the seventh day of storage at 8 °C. At the same time, a significant decrease in the H_4_folate content (31%) was found. The determined folate losses in FM 4 on days 14 and 21 of refrigerated storage did not exceed 15% and 48%, respectively, and were the lowest compared to other fermented milks at the same storage time. During the refrigerated storage of FM 1–3, a significant decrease (*P* < 0.05) in the folate content was observed on the seventh day of cold storage. The lowest losses of 27% after day 7 were observed in the product FM 3. On day 14 in FMs 1–3, folate losses were 50%, and on day 21 exceeded 65% in both FM 2 and FM 3. When comparing the stability of the two identified folate forms in the tested fermented milk samples during storage at 8 °C, only in FM 2 was clearly higher stability of H_4_folate noted. The losses during storage ranged from 23% to 25% compared to 59–75% losses of the methyl form. The observed folate losses in the current experiment during storage may result from the acid pH of fermented foods, in which most folate vitamers are less stable and because folate was consumed by bacteria [9,12].

Although yeasts are well known for their ability to produce folate during the fermentation process, the capacity of LAB for folate production is not yet clear. The reason is that both folate production and consumption are observed in culture media and in fermented foods [9,12]. Moreover, as noted by Saubade et al. [9], it is sometimes impossible to compare data on folate production by LAB in different studies due to the differences in methods applied and units in which results are expressed. In some studies, the authors measured the folate increase in the cell biomass, while other studies measured the folate increase in the supernatant of the culture medium. Furthermore, most studies in this field have tested the synthesis of folate by LAB in culture media. Although this is helpful in understanding the influence of different parameters, it may not be suitable for selecting LAB strains that increase folate amounts in real food matrices [9]. Table 2 shows the folate amounts determined by other authors and in own studies in milk fermented with LAB, by single strains or in combination.

The authors emphasize that folate synthesis by LAB depends on many factors, including the strain, cultivation conditions, incubation time, the composition of the culture medium, and the presence of folate precursors or prebiotic supplementation [9,14]. Nevertheless, depending on applied starter cultures and storage conditions, the folate concentration in fermented milk can be increased to more than 150 µg/L [46,58]. In addition, as shown in Table 2, the use of a combination of different LAB may be more efficient than the use of single cultures [9]. Crittenden et al. [17] found that higher folate amounts were observed in milk fermented with a co-culture of *Streptococcus thermophilus and Bifidobacterium animalis* than in single cultures. Laiño et al. [58] noted that among different combinations, a strain of *Lactobacillus delbruecki* subsp. *bulgaricus* and *Streptococcus thermophilus* gave the best results in milk. In turn, Ayad [59] showed that the combination of *Lactococcus lactis* subsp. *lactis* and *Lactococcus lactis* subsp. *cremoris* was the most effective in folate production in Domiati cheese. In the authors’ own study, the highest results were obtained with the mix of *Lactobacillus* species and *Streptococcus thermophilus* and *Bifidobacterium bifidum*.

However, the folate content of fermented milk enriched by LAB, including *Bifidobacteria*, is generally still too low to significantly contribute to the folate requirements at a satisfactory level. Most of the values presented in Table 2 of the coverage of the daily demand for folate by eating fermented milk products are not realistic. Even in the case of the tested FM 3 (with the highest folate content of 105 µg/kg), up to one kilogram of the product would have to be eaten during the day to cover approximately 25% of the daily requirement for this vitamin. Although LAB has been reported to significantly increase folate content in fermented products, it is still not enough to cover the daily requirements for this vitamin. Therefore, other solutions are needed to increase the folate content in foods fermented with LAB [9]. One option might be the combination of LAB with yeasts, which were reported to be more efficient in folate production when compared to LAB [52,61]. Another option, suggested by Saubade et al. [9], is to use LAB strains able to produce folate vitamers (such as 5-CH_3_-H_4_folate) which are generally more stable at low pH, which is the main folate form in fermented dairy products.

### 2.2. Fatty Acid Composition

Changes in the content of each group of fatty acids during storage at 8 °C in fermented milks were observed (Table 3). In all analyzed samples, fresh and stored, saturated fatty acids (SFA) were dominant. The mean content of SFA in the analyzed fermented milk inoculated with *Lactobacillus delbrueckii* subsp. *bulgaricus* and *Streptococcus thermophilus* (FM 1) was the lowest in fresh products. The fermented milk analyzed after 7, 14, and 21 days of storage was characterized by a significantly higher (*P* < 0.05) content of these acids. In stored fermented milk inoculated with *Lactococcus lactis* subsp. *cremoris*, *Leuconostoc*, *Lactococcus lactis* subsp. *lactis* and *Lactococcus lactis* subsp. *lactis diacetylactis* (FM 2) and *Lactobacillus delbrueckii* subsp. *bulgaricus*, and *Lactobacillus delbrueckii* subsp. *lactis Streptococcus thermophilus* and *Bifidobacterium bifidum* (FM 3), the mean content of SFA fluctuated. In fermented milk produced with *Lactobacillus delbrueckii* subsp. *Bulgaricus*, *Streptococcus thermophilus*, and *Bifidobacterium bifidum* (FM 4), the highest content of these acids was found in fresh products. Significantly lower (*P* < 0.05) SFA content was found in stored fermented milk. The content of PUFA (polyunsaturated fatty acids) in all analyzed products was at a similar level. In the case of MUFA (monounsaturated fatty acids) content, only in fermented milk inoculated with *Lactobacillus delbrueckii* subsp. *bulgaricus* and *Streptococcus thermophilus* (FM 1) and *Lactobacillus delbrueckii* subsp. *Bulgaricus*, *Streptococcus thermophilus*, and *Bifidobacterium bifidum* (FM 4) did not change significantly during storage. The contents of short-chain fatty acids (SCFA) in fermented milk inoculated with *Lactobacillus delbrueckii* subsp. *Bulgaricus*, *Streptococcus thermophilus*, and *Bifidobacterium bifidum* (FM 4) fluctuated during the storage period. In other analyzed fermented milks, the content of these acids changed significantly (*P* < 0.05) (Table 3).

*n*-3 and *n*-6 polyunsaturated fatty acids are essential nutrients that cannot be synthesized in the body and must be obtained from the diet. It is important for the content of these acids to remain stable or increase during storage conditions to provide their beneficial effects. The results presented in Table 3 indicate that the cold storage time did not cause significant changes in the content of *n*-3 and *n*-6 acids. Only fluctuations in the content of these acids in stored fermented milks were observed. However, in a previous study [62], a significant decrease in *n*-3 and *n*-6 polyunsaturated fatty acid contents in cow milk yogurts produced with a starter culture containing *Streptococcus thermophilus* and *Lactobacillus delbrueckii* subsp. *bulgaricus* was observed during storage.

Research has shown that in stored fermented milk produced with the combination of *Lactococcus lactis* subsp. *cremoris*, *Leuconostoc*, *Lactococcus lactis* subsp. *lactis* and *Lactococcus lactis* subsp. *lactis diacetylactis* (FM 2) and *Lactobacillus delbrueckii* subsp. *bulgaricus*, *Streptococcus themophilus*, and *Bifidobacterium bifidum* (FM 4), significant changes (*P* < 0.05) in the *n*-6/*n*-3 acid ratio were observed. In other analyzed fermented milks, the ratio of these acids fluctuated slightly (Table 3). The proportions of specific groups of fatty acids in products are of special importance from a nutritional perspective. Excessive amounts of *n*-6 polyunsaturated fatty acids (PUFA) and a very high *n*-6/*n*-3 ratio promote the pathogenesis of many diseases, whereas increased levels of *n*-3 PUFA (a low *n*-6/*n*-3 ratio) exert suppressive effects [63,64].

The CLA content in fresh fermented milk ranged from 2.54 mg/g fat in FM 1 inoculated with *Lactobacillus delbrueckii* subsp. *bulgaricus* and *Streptococcus thermophilus* to 3.61 mg/g fat in FM2 produced with the combination of *Lactococcus lactis* subsp. *cremoris*, *Leuconostoc*, *Lactococcus lactis* subsp. *lactis*, and *Lactococcus lactis* subsp. *lactis diacetylactis* (Table 3). In all tested fermented milks, the time of refrigerated storage caused changes in CLA content, and the highest levels of this compound were found in all fermented milks stored for 21 days at 8 °C, with the highest amount of 3.93 mg/g fat in FM 2. However, in FM 1 and FM 2, the changes in *cis9trans11* C18:2 acid content after 7, 14, and 21 days were not significantly different (*P* < 0.05) compared to the CLA content in fresh fermented samples. Instead, in fermented milks enriched with *Bifidobacterium bifidum*, a significant increase in CLA contents was observed.

In FM 3 (with a mixture of *Lactobacillus delbrueckii* subsp. *bulgaricus*, *Lactobacillus delbrueckii* subsp. *lactis*, *Streptococcus thermophilus*, and *Bifidobacterium bifidum*), a significant increase was noted after 14 and 21 days of cold storage, while in FM 4 (inoculated with *Lactobacillus delbrueckii* subsp. *bulgaricus*, *Streptococcus thermophilus*, and *Bifidobacterium bifidum*) it was noted after 21 days. The obtained results demonstrate the potential of commercially available vaccines of LAB cultures, with special emphasis on *Bifidobacterium* to synthesize CLA during refrigerated storage. Research by other authors confirmed that the type of applied starter culture and storage time affects the content of CLA in fermented milk. According to a study by Domagała et al. [42], one of the seven starter cultures used by these authors (a yogurt culture ABY-2) caused an increase in the CLA content in stored fermented cream. Changes in CLA content in yogurts produced from cow’s milk stored for 14 days at 5 °C were also demonstrated by Serafeimidow et al. [65]. According to their research, after seven days of storage, the yogurts from cow’s milk were characterized by a higher content of CLA than the products analyzed on day 1. Significantly lower content of this acid was found by these authors in yogurts analyzed after 14 days of storage. Changes in CLA content in ecological and conventional fermented milk stored for seven days at 4 °C were also reported by Florence et al. [66]. According to Paszczyk et al. [67], out of three starter cultures used in the study, only one culture, Ceska-star Y508 (*Lactobacillus delbrueckii* subsp. *bulgaricus* and *Streptococcus thermophiles*), caused a significant increase in CLA content in the stored fermented milk drinks.

## 3. Materials and Methods

### 3.1. Samples

The research material was fermented milk (FM) from four productions. The FM covered by the study was produced using selected starter cultures. Four batches of fermented milk were produced using different selected starter cultures. Analyses were carried out for freshly produced FM samples and for samples stored at 8 ± 1 °C for 7, 14, and 21 days. Fermented milk was produced with the thermostat method according to the following technological scheme: raw milk was heated to 45 °C, centrifuged and degassed (80 kPa; 60 °C), then subjected to HTST pasteurization (72 °C/15 s; ALFA-LAVAL P20-HB pasteurizer, Lund, Sweden) and cooled to 6 °C. Afterward, it was normalized to a fat content of 2 ± 0.1% (addition of skim milk) and subjected to two-stage homogenization (18/5 MPa, 65 °C; homogenizer CN003, Spomasz Bełżyce, Poland) and long-term VHT pasteurization (90 °C/5 min; ALFA-LAVAL P20-HB pasteurizer, Lund, Sweden). After cooling to 45 °C, the milk was inoculated with pre-incubated for 2 h in 45 °C four different starter cultures (powder form) in the amount of 1 mL/L of milk. FM 1 was inoculated with FD-DVS YC-380 Yo-Flex containing *Lactobacillus delbrueckii* subsp. *bulgaricus* and *Streptococcus thermophilus* (Chr. Hansen, Hørsholm, Denmark). FM 2 was inoculated with FD-DVS FLORA DANICA starter culture containing *Lactococcus lactis* subsp. *cremoris*, *Leuconostoc*, *Lactococcus lactis* subsp. *lactis*, and *Lactococcus lactis* subsp. *lactis diacetylactis* (Chr. Hansen, Hørsholm, Denmark); FM 3 was inoculated with FD-DVS YC-180 Yo-Flex and BB-12 starter culture containing *Lactobacillus delbrueckii* subsp. *bulgaricus*, *Lactobacillus delbrueckii* subsp. *lactis*, *Streptococcus thermophilus*, and *Bifidobacterium bifidum*. FM 4 was inoculated with YC-X16 and BB-12 starter culture containing *Lactobacillus delbrueckii* subsp. *bulgaricus*, *Streptococcus thermophilus*, and *Bifidobacterium bifidum* (Chr. Hansen, Hørsholm, Denmark). The fermented milk drinks produced with the addition of selected starter cultures were transferred to unitary packages and left to ripen in thermostats (Binder GF115, Tuttlingen, Germany) at 43.5 °C until they reached pH 4.6 (c.a. 5 h).

### 3.2. Folate Analysis

#### 3.2.1. Chemicals, Enzymes, and Standards

Water was purified in the Mili-Q system (Millipore; Vienna, Austria), acetonitrile was of HPLC grade, and the other chemicals were of analytical grade. Protease (E.C.3.4.24.31), obtained from Sigma Aldrich (St. Louis, MO, USA), was dissolved in 0.1 M phosphate buffer, pH 7.0, with 1% (*w*/*v*) sodium ascorbate and 0.1% (*v*/*v*) 2-mercaptoethanol (in the amount of 4 mg/mL) just before the analysis to avoid bacterial contamination, which can synthesize folate during incubation. Fresh rat plasma, used as a folate conjugase source, was purchased from Europa Bioproducts Ltd. (Cambridge, Great Britain) and prepared according to Patring et al. [68].

Folate standards: folic acid, 5-methyltetrahydrofolate (5-CH_3_-H_4_folate), 5-formyltetrahydrofolate (5-HCO-H_4_folate), and tetrahydrofolate (H_4_folate) were obtained from Sigma Aldrich (St. Louis, MO, USA). 10-formyl folic acid (10-HCO-folic acid) and 5,10-methenyltetrahydrofolate (5,10-CH^+^-H_4_folate) were obtained from Schircks Laboratories (Jona, Switzerland). All standards were prepared as described by Konings [69]. 10-formyldihydrofolate (10-HCO-H_2_folate) was obtained from 5,10-CH^+^-H_4_folate according to Pfeiffer et al. [70].

#### 3.2.2. Sample Preparation

The content of folate vitamers was analyzed in triplicate using the sample pre-treatment method described by Gujska et al. [47]. During sample preparation, folates were protected against oxidation by carrying out the analysis under dim light and cooling the samples in ice after heating. Briefly, 10 g (accurate to 0.001 g) of fermented milk sample was inserted into a 30 mL PPCO Oak Ridge PPCO centrifuge tube (Nalgene; Rochester, NY, USA). Following this, 15 mL of an extraction buffer (0.1 M phosphate buffer, pH 7.0, with 1% (*w*/*v*) sodium ascorbate and 0.1% (*v*/*v*) 2-mercaptoethanol) were added. Samples were shaken (2500 rpm/10 s IKA Vortex 4 basic; Staufen, Germany) for 1 min and then transferred into a boiling water bath, heated for 15 min, shaken three times, and then cooled in ice. A total of 1 mL of the protease solution (4 mg/mL) and 0.25 mL of rat plasma conjugase were then added to each sample, and the samples were incubated at 37 °C for 4 h (POL-EKO; Rybnik, Poland). During incubation, samples were subjected to mild stirring (using a magnetic stirrer). Following this, they were heated in a boiling water bath for 5 min to inactivate enzymes, then cooled in ice and then centrifuged twice at 12,000 rpm/4 °C/20 min (MPW-350R; Warsaw, Poland). Each time, supernatants were collected in 50 mL amber volumetric flasks, which were filled up with the extraction buffer. The extract was filtered through the filter paper into amber glass bottles, flushed with nitrogen, and stored at −70 °C until the HPLC analysis.

Prior to the HPLC analysis, the samples were purified using Solid Phase Extraction (SPE) on Strong Anion Exchange (SAX) Bakerbond SPE JT cartridges (3 mL × 500 mg Solid Phase Extraction Column, PP (polypropylene), Quaternary Amine (N^+^) Anion Exchange; Philipsburg, MT, USA) as described by Jastrebova et al. [71].

#### 3.2.3. Folate Quantification

The chromatographic separation of folates was carried out according to Czarnowska and Gujska [72] using the HPLC system (Shimadzu Series LC-10A, Kyoto, Japan) and the C18 LC column: Synergi 4u Hydro-RP 80 Å (250 × 4.6 mm^2^, 4 µm; Phenomenex; Torrance, CA, USA). The total separation time was 41 min. The chromatographic conditions for gradient elution were as follows: flow rate: 1 mL/min, volume injection 50 mL, column temperature 25 °C, UV detection: 290 nm; fluorescence detection: 290 nm excitation and 360 nm emission for 5-CH_3_-H_4_folate, 5-HCO-H_4_folate, and H_4_folate; 360 nm excitation and 460 nm emission for 10-HCO-folic acid. The mobile phase was acetonitrile with a 30 mM phosphoric acid buffer (pH 2.3). The gradient started at 5% acetonitrile and remained at that level for the first 8 min before being raised to 17.5% within 17 min. Peaks were identified based on the retention times of standards. Quantification of the identified individual folate vitamers was based on fluorescence detection using the external multilevel (*n* = 8) calibration curves. The results of folate vitamer content determination in the tested fermented milk were based on the fresh weight and presented as means with standard deviations from triplicates. The total folate content was the sum of 5-CH_3_-H_4_folate and H_4_folate contents expressed as folic acid content using the molar absorption coefficient given by Blakely [73]. Differences in the mean total folate content in fresh and stored fermented milk samples were compared using the Duncan multiple range test, with a significance level of *P* < 0.05. The statistical analysis was carried out using Statistica software, version 13.1 (StatSoft; Cracow, Poland) [74].

### 3.3. Fatty Acid Composition

#### 3.3.1. Fat Extraction

Fat was isolated from the analyzed fermented milks with the method of Folch et al. [75] with some modifications. Briefly, yogurts were heated to the temperature of 20 °C and thoroughly mixed. Approximately 10 g of samples (0.01 g) were homogenized (IKA Ultra-Turrax^®^ T18 digital, Staufen, Germany) for 1 min with 100 mL of methanol. Next, 100 mL chloroform was added and homogenized for 2 min. The prepared mixture was filtered to a 500 mL glass cylinder. The solid residue was mixed in 200 mL chloroform: methanol (2:1 *v*/*v*) and homogenized again for 3 min. The mixture was transferred to the same cylinder. Then, 0.88% sodium chloride in water was added to the total filtrate (in the amount constituting 1/4 volume of filtrate); it was shaken vigorously for 1 min and left overnight to separate the layers. Next, the upper layer was removed using a water pump, and the lower layer was washed twice with a water–methanol mixture (1:1 *v*/*v*) and was filtered through anhydrous (VI) sodium sulfate. The solvent was evaporated. Methyl esters were prepared from the separated fat.

#### 3.3.2. Preparation of Fatty Acid Methyl Esters

Fatty acid methyl esters were prepared according to the IDF method using a methanolic solution of KOH (ISO 15884:2002) [76]. N-hexane and 2 M KOH in methanol were added to each fat sample, and the mixture was then shaken. Sodium hydrogen sulfate (NaHSO_4_) was then added, and the mixture was centrifuged (3000 rpm). The top layer of prepared methyl esters was collected for chromatographic analysis.

#### 3.3.3. Gas Chromatography (GC) Analysis

The fatty acid contents were determined with the GC-FID method using the following: a capillary column (100 m × 0.25 mm i.d., film thickness 0.20 μm) (Chrompack, Middelburg, the Netherlands) with a CP Sil 88 stationary phase, and helium applied as a carrier gas at the flow rate of 1.5 mL/min. Sample injection volume was 0.4 μL (split: 50:1). Determinations were carried out under the following conditions: column temp. 60 °C (1 min)–180 °C, Δt = 5 °C/min, detector and injection temperatures of 250 °C and 225 °C, respectively.

#### 3.3.4. Identification and Calculation of Fatty Acids

Identification of fatty acids was carried out based on a comparison of their retention time with the retention time of methyl esters of fatty acids of the reference milk fat (BCR Reference Materials) of the CRM 164 symbol and literature data [76,77,78,79]. The *cis*9*trans*11 C18:2 (CLA) isomer was identified using a mixture of CLA methyl esters (Sigma-Aldrich, Germany). The contents of fatty acids were calculated in mg/g fat according to the applicable standard (methyl ester of C21:0 acid, Sigma-Aldrich, Germany). The statistical analysis of results was carried out using Statistica version 13.1 (StatSoft; Cracow, Poland) [74] software based on a one-way analysis of variance (ANOVA) at a significance level at *P* < 0.05. The differences between mean values were evaluated using Duncan’s test.

## 4. Conclusions

Many studies have presented promising results for the use of lactic acid bacteria to synthesize folate during fermentation. However, in other studies (including the authors’ own study), either folate consumption by LAB or low folate production was observed. Commercial yogurts eaten in a normal daily portion cannot meet 10–20% of the daily recommended intake.

Combining LAB with different abilities to improve nutritional food quality is an option to maximize beneficial properties. Bio-fortified fermented milks offer a good alternative to develop a functional fermented food with increased amounts of essential compounds. There is an emerging opportunity for the food industry to use selected strains as starter cultures able to synthesize compounds such as vitamins and conjugated linoleic acid with a number of pro-health properties. For this reason, further research should focus on careful testing and selecting LAB strains able to produce folates, *cis*9*trans*11 C18:2 acid in high amounts, and keeping these nutrients stable during storage. Moreover, research is also required on the design and optimization of favorable conditions for such production.

## Figures and Tables

**Table 1:** Folate content and losses in fresh and refrigerated fermented milk.

Fermented Milk (FM)	Days of Storage at 8 °C	H4folate (µg/kg)	5-CH3-H4folate(µg/kg)	Total Folates (Sum as Folic Acid) (µg/kg)	Folates Losses during Storage (%)
FM 1 *Lactobacillus delbrueckii* subsp. *bulgaricus*, and *Streptococcus thermophilus*	0	22.1 ^1^ ± 1.1	24.4 ± 1.3	45.3 ^a2^ ± 0.3	-
	7	12.3 ± 0.8	15.2 ± 0.8	27.1 ^b^ ± 0.8	40
	14	8.4 ± 0.2	14.2 ± 0.5	22.3 ^c^ ± 0.8	50
	21	11.3 ± 0.6	10.3 ± 0.3	20.3 ^d^ ± 0.9	55
FM 2 *Lactococcus lactis* subsp. *cremoris*, *Leuconostoc*, *Lactococcus lactis* subsp. *lactis*, and *Lactococcus lactis* subsp. *lactis diacetylactis*	0	12.2 ± 0.2	61.2 ± 3.1	71.1 ^a^ ± 3.4	-
	7	9.4 ± 0.4	25.2 ± 2.2	33.2 ^b^ ± 2.1	53
	14	12.3 ± 0.1	20.0 ± 2.4	31.2 ^b^ ± 2.3	56
	21	9.2 ± 0.2	15.3 ± 1.1	23.3 ^c^ ± 1.1	67
FM 3 *Lactobacillus delbrueckii* subsp. *bulgaricus*, *Lactobacillus delbrueckii* subsp. *lactis*, *Streptococcus thermophilus*, and *Bifidobacterium bifidum*	0	27.5 ± 2.3	84.0 ± 2.7	105.4 ^a^ ± 6.1	-
	7	17.8 ± 1.1	63.3 ± 0.9	77.2 ^b^ ± 3.6	27
	14	15.2 ± 0.8	34.8 ± 3.2	49.4 ^c^ ± 4.8	53
	21	11.3 ± 0.6	19.2 ± 1.8	30.3 ^d^ ± 2.6	71
FM 4 *Lactobacillus delbrueckii* subsp. *bulgaricus*, *Streptococcus thermophilus*, and *Bifidobacterium bifidum*	0	23.4 ± 0.2	41.1 ± 2.1	60.3 ^b^ ± 1.4	-
	7	16.2 ± 0.7	65.4 ± 5.2	79.2 ^a^ ± 6.3	+31
	14	13.3 ± 1.4	39.0 ± 3.3	51.1 ^c^ ± 4.1	15
	21	8.4 ± 0.6	23.1 ± 2.4	31.2 ^d^ ± 2.1	48

^1^ The results are presented on the fresh weight as the mean of three replicates ± standard deviation. ^2^ Means in the column, for each fermented milk with the same letter, are not significantly different at *P* < 0.05.

**Table 2:** Folate content determined in fermented milk inoculated with LAB.

Folate Content ^1^ in Fermented Milk	Coverage of the Daily Demand for Folate (%) ^2^	Producing Microorganisms	Ref.
5–50 µg/kg	1–12	*S. thermophilus*	[50]
20–50 µg/L	5–12	*S. thermophilus*	[55]
50–200 µg/kg	12–50	*S. thermophilus*	[56]
250–280 µg/L	63–70	*L. amylovorus*, *S. thermophilus* and *L. delbruecki* subsp. *bulgaricus*	[46]
10–70 µg/L	3–18	*L. delbruecki* subsp. *bulgaricus*	[57]
80–180 µg/L	20–45	*L. delbruecki* subsp. *bulgaricus*, *S. thermophilus*	[58]
27–35 µg/kg	7–9	*L. delbruecki* subsp. *bulgaricus*, *S. thermophilus*	[47]
45 µg/kg	11	*L. delbruecki* subsp. *bulgaricus*, *S. thermophilus*	Own study
50–100 µg/L	13–25	*B. longum*	[16]
60–90 µg/kg	15–23	*S. thermophilus*, *B. animalis*	[17]
30–60 µg/kg	8–15	*S. thermophilus*, *B. longum*	[49]
60	15	*L. delbrueckii* subsp. *bulgaricus*, *S. thermophilus*, and *B. bifidum*	Own study
105 µg/kg	26	*L. delbruecki* subsp. *bulgaricus*, *L. lactis*, *S. thermophilus*, and *B. bifidum*	Own study
120–130 µg/kg	30–33	*Lc. lactis* subsp. *lactis*, *Lc.* *lactis* subsp. *cremoris*	[59]
2–20 µg/L	1–5	*Lc. lactis* subsp. *cremoris*	[53]
71 µg/kg	18	*Lc. lactis* subsp. *cremoris*, *Leuconostoc*, *Lc. lactis* subsp. *lactis*, and *L. lactis* subsp. *lactis diacetylactis*	Own study

^1^ Folate content expressed in fresh weight basis. ^2^ Coverage of the daily demand for folate by adults, based on RDA (recommended daily allowance) of 400 µg set for men and women above 19 years old, by eating 1 kg or 1 L of the fermented milk product [60].

**Table 3:** The content of fatty acid groups in fresh and refrigerated fermented milks (mg/g fat).

Fermented Milk (FM)	Fatty Acids	Days of Storage at 8 °C			
		0	7	14	21
FM 1 *Lactobacillus delbrueckii* subsp. *bulgaricus* and *Streptococcus thermophilus*	SCFA ^1^	98.58 ± 8.34 ^a^	74.76 ± 4.16 ^b^	68.97 ± 7.79 ^b^	94.02 ± 9.97 ^a^
	SFA	481.28 ± 17.06 ^b^	541.03 ± 20.45 ^a^	546.80 ± 22.96 ^a^	562.27 ± 19.44 ^a^
	MUFA	58.33 ± 5.39 ^a^	53.81 ± 2.73 ^a^	56.48 ± 5.06 ^a^	58.30 ± 8.41 ^a^
	PUFA	21.78 ± 2.40 ^a2^	20.26 ± 2.44 ^a^	21.29 ± 2.63 ^a^	22.27 ± 3.05 ^a^
	*n*-3	2.05 ± 0.01 ^a^	2.03 ± 0.11 ^a^	2.14 ± 0.18 ^a^	2.37 ± 0.39 ^a^
	*n*-6	15.21 ± 0.27 ^a^	15.25 ± 0.63 ^a^	15.93 ± 0.88 ^a^	16.76 ± 2.60 ^a^
	*n*-6/*n*-3	7.41 ± 0.14 ^a^	7.53 ± 0.12 ^a^	7.47 ± 0.25 ^a^	7.07 ± 0.11 ^a^
	CLA	3.51 ± 0.22 ^a^	3.38 ± 0.11 ^a^	3.59 ± 0.31 ^a^	3.73 ± 0.67 ^a^
FM 2 *Lactococcus lactis* subsp. *cremoris*, *Leuconostoc*, *Lactococcus lactis* subsp. *lactis*, and *Lactococcus lactis* subsp. *lactis diacetylactis*	SCFA	99.92 ± 2.92 ^a^	96.31 ± 13.94 ^a^	73.51 ± 7.24 ^b^	83.12 ± 5.47 ^b^
	SFA	489.77 ± 9.15 ^a^	496.57 ± 25.64 ^a^	477.98 ± 20.01 ^a^	502.29 ± 8.33 ^a^
	MUFA	52.44 ± 3.21 ^c^	58.13 ± 3.03 ^b^	53.40 ± 3.88 ^bc^	63.49 ± 3.71 ^a^
	PUFA	20.17 ± 2.58 ^a^	19.03 ± 2.03 ^a^	18.70 ± 2.17 ^a^	20.73 ± 3.95 ^a^
	*n*-3	2.03 ± 0.03 ^a^	2.05 ± 0.03 ^a^	2.06 ± 0.02 ^a^	2.06 ± 0.03 ^a^
	*n*-6	13.97 ± 0.60 ^a^	12.97 ± 0.47 ^a^	12.90 ± 0.49 ^a^	13.86 ± 1.01 ^a^
	*n*-6/*n*-3	6.87 ± 0.39 ^a^	6.32 ± 0.14 ^b^	6.25 ± 0.30 ^b^	6.71 ± 0.40 ^ab^
	CLA	3.61 ± 0.17 ^ab^	3.84 ± 0.16 ^a^	3.43 ± 0.38 ^b^	3.93 ± 0.09 ^a^
FM 3 *Lactobacillus delbrueckii* subsp. *bulgaricus*, *Lactobacillus delbrueckii* subsp. *lactis*, *Streptococcus thermophilus*, and *Bifidobacterium bifidum*	SCFA	67.96 ± 5.52 ^c^	65.32 ± 6.94 ^c^	78.14 ± 4.27 ^b^	88.37 ± 3.49 ^a^
	SFA	607.54 ± 20.84 ^a^	468.87 ± 12.05 ^b^	478.63 ± 9.23 ^b^	612.00 ± 25.36 ^a^
	MUFA	52.15 ± 13.09 ^ab^	43.24 ± 4.99 ^b^	48.79 ± 2.76 ^b^	62.75 ± 2.50 ^a^
	PUFA	18.02 ± 4.60 ^a^	16.59 ± 1.40 ^a^	17.63 ± 2.85 ^a^	17.83 ± 2.00 ^a^
	*n*-3	1.86 ± 0.57 ^a^	1.65 ± 0.22 ^a^	1.80 ± 0.11 ^a^	1.68 ± 0.12 ^a^
	*n*-6	13.70 ± 3.98 ^a^	12.29 ± 1.11 ^a^	13.35 ± 1.04 ^a^	12.76 ± 0.46 ^a^
	*n*-6/*n*-3	7.39 ± 0.16 ^a^	7.49 ± 0.39 ^a^	7.43 ± 0.32 ^a^	7.63 ± 0.78 ^a^
	CLA	2.54 ± 0.28 ^c^	2.71 ± 0.30 ^bc^	3.06 ± 0.18 ^ab^	3.42 ± 0.23 ^a^
FM 4 *Lactobacillus delbrueckii* subsp. *bulgaricus*, *Streptococcus thermophilus*, and *Bifidobacterium bifidum*	SCFA	70.46 ± 15.24 ^a^	64.76 ± 4.16 ^a^	74.27 ± 7.45 ^a^	75.02 ± 4.19 ^a^
	SFA	570.24 ± 9.96 ^a^	538.53 ± 22.74 ^a^	563.05 ± 22.10 ^a^	567.52 ± 24.15 ^a^
	MUFA	52.15 ± 16.95 ^a^	53.81 ± 2.73 ^a^	56.48 ± 5.06 ^a^	58.30 ± 8.41 ^a^
	PUFA	18.07 ± 4.58 ^a^	20.26 ± 2.44 ^a^	20.04 ± 2.67 ^a^	20.82 ± 2.08 ^a^
	*n*-3	1.86 ± 0.53 ^a^	2.03 ± 0.11 ^a^	2.14 ± 0.18 ^a^	2.24 ± 0.13 ^a^
	*n*-6	13.59 ± 3.93 ^a^	15.25 ± 0.63 ^a^	15.68 ± 0.69 ^a^	15.44 ± 0.65 ^a^
	*n*-6/*n*-3	7.33 ± 0.15 ^ab^	7.53 ± 0.12 ^a^	7.36 ± 0.46 ^a^	6.89 ± 0.28 ^b^
	CLA	2.88 ± 0.65 ^b^	3.38 ± 0.11 ^ab^	3.59 ± 0.31 ^ab^	3.89 ± 0.65 ^a^

^1^ SCFA—short-chain fatty acids (C4–C10); SFA—saturated fatty acids; MUFA—monounsaturated fatty acids; PUFA—polyunsaturated fatty acids, CLA (*cis*9*trans*11 C18:2)—conjugated linoleic acid. ^2^ Mean values, for fresh and stored fermented milk, in rows with the same letter are not significantly different at *P* < 0.05.

## Data Availability

Data is contained within the article.

## Sample Availability

Samples of the compounds are available from the authors.
